# Analysis of the *ANA* gene as a candidate for the chromosome 21q oral cancer susceptibility locus

**DOI:** 10.1054/bjoc.2000.1656

**Published:** 2001-03

**Authors:** N Yamamoto, K Uzawa, T Yakushiji, T Shibahara, H Noma, H Tanzawa

**Affiliations:** First Department of Oral and Maxillo-Facial Surgery, Tokyo Dental College, 1-2-2 Masago, Mihama-ku, Chiba, 261-8502, Japan; Department of Oral Surgery, Chiba University School of Medicine, 1-8-1 Inohana, Chuo-ku, Chiba, 260-8670, Japan

**Keywords:** oral cancer, *ANA* gene, loss of heterozygosity, chromosome 21, DNA methylation

## Abstract

Loss of heterozygosity (LOH) on the long arm of chromosome 21 (21q) is observed in several human malignancies. We identified novel tumour suppressor loci on this region in primary oral squamous cell carcinomas (OSCCs). To further determine the role of 21q deletions in oral cavity tumorigenesis, 63 OSCCs were examined for LOH at 21q using 7 microsatellite markers. LOH was observed in 32 of 63 cases (50.8%) that were informative for at least one of the loci analysed. Two distinct deleted regions were identified at chromosomal region 21q11.1. The possible involvement of *ANA* (abundant in neuroepithelium area), a candidate tumour suppressor gene (TSG) located on 21q11.2–21.1, was also evaluated for 20 OSCCs and 9 OSCC-derived cell lines. 60% of tumours (12/20) and 88.9% (8/9 cell lines) showed absent or reduced mRNA gene expression; only one OSCC case had a nucleotide substitution in the *ANA* gene. Interestingly, the frequency of the suppressed *ANA* mRNA expression was greater in stage IV tumours than in earlier stages. In addition, re-expression of the *ANA* gene mRNA was induced in 4 cell lines after treatment with 5-aza-2′-deoxycytidine, a DNA demethylating agent. These findings demonstrate that there may be at least 2 distinct TSGs on 21q11.1; loss of *ANA* gene expression could be involved in the progression of human OSCC; and aberrant methylation of the *ANA* gene promoter may participate in the transcriptional silencing of the gene in oral cancer cells. © 2001 Cancer Research Campaign http://www.bjcancer.com


British Journal of Cancer (2001) 84(6), 754–759
© 2001 Cancer Research Campaign
doi: 10.1054/ bjoc.2000.1656, available online at http://www.idealibrary.com on IDEAL®

http://www.bjcancer.com

### Analysis of the *ANA* gene as a candidate for the chromosome 21q oral cancer susceptibility locus

**N Yamamoto[1], K Uzawa[2], T Yakushiji[1], T Shibahara[1], H Noma[1] and H Tanzawa[2]**

[1]First Department of Oral and Maxillo-Facial Surgery, Tokyo Dental College, 1-2-2 Masago, Mihama-ku, Chiba 261-8502, Japan; [2]Department of Oral Surgery, Chiba University School of Medicine, 1-8-1 Inohana, Chuo-ku, Chiba 260-8670, Japan

**Summary** Loss of heterozygosity (LOH) on the long arm of chromosome 21 (21q) is observed in several human malignancies. We identified novel tumour suppressor loci on this region in primary oral squamous cell carcinomas (OSCCs). To further determine the role of 21q deletions in oral cavity tumorigenesis, 63 OSCCs were examined for LOH at 21q using 7 microsatellite markers. LOH was observed in 32 of 63 cases (50.8%) that were informative for at least one of the loci analysed. Two distinct deleted regions were identified at chromosomal region 21q11.1. The possible involvement of *ANA* (abundant in neuroepithelium area), a candidate tumour suppressor gene (TSG) located on 21q11.2–21.1, was also evaluated for 20 OSCCs and 9 OSCC-derived cell lines. 60% of tumours (12/20) and 88.9% (8/9 cell lines) showed absent or reduced mRNA gene expression; only one OSCC case had a nucleotide substitution in the *ANA* gene. Interestingly, the frequency of the suppressed *ANA* mRNA expression was greater in stage IV tumours than in earlier stages. In addition, re-expression of the *ANA* gene mRNA was induced in 4 cell lines after treatment with 5-aza-2′-deoxycytidine, a DNA demethylating agent. These findings demonstrate that there may be at least 2 distinct TSGs on 21q11.1; loss of *ANA* gene expression could be involved in the progression of human OSCC; and aberrant methylation of the *ANA* gene promoter may participate in the transcriptional silencing of the gene in oral cancer cells. © 2001 Cancer Research Campaign http://www.bjcancer.com

**Keywords**: oral cancer; *ANA* gene; loss of heterozygosity; chromosome 21; DNA methylation

Oral cancer is one of the most common malignancies worldwide. Although squamous cell carcinoma (SCC) contributes to over 90% of all malignant tumours of the oral cavity, there is no relative tool to identify cells possessing the cancerous phenotype within a normal squamous cell population.

It has been generally accepted that loss of function of the tumour suppressor gene(s) (TSG) is a key event during the progression of human malignancies (Fearon, 1998). Investigators have identified genetic alterations associated with oral squamous cell carcinomas (OSCCs), such as mutations in the *p53* gene (Chiba et al, 1996), the *APC* gene (Largery et al, 1994; Uzawa et al, 1994), and mutation or hypermethylation of the *p16* gene (Heinzel et al, 1996; Miracca et al, 1999), as well as allelic imbalances including genetic instability (Ishwad et al, 1995; Ogawara et al, 1998) and loss of heterozygosity (LOH) on several chromosomes (Maestro et al, 1993; Uzawa et al, 1996; Wu et al, 1997; Wang et al, 1999). In addition, previous allelotyping studies of human head and neck SCC (HNSCC) have shown multiple chromosomal regions in which LOH frequently was observed (Ah-See et al, 1994; Field et al, 1996; Scully and Field, 1997). This evidence indicates that there are a number of TSGs involved in the carcinogenesis of HNSCC, including OSCC.

In simple allelotype studies, LOH at a marker on 21q was observed in 37% of oesophageal SCCs (Aoki et al, 1994) and in 50% of SCCs of the lung (Sato et al, 1994). Our deletion mapping study demonstrated that at least 3 distinct TSGs associated with OSCCs may be harboured on 21q (Yamamoto et al, 1999). Recently, a candidate TSG, abundant in neuroepithelium area gene (*ANA*), was identified and mapped to 21q11.2–q21.1 (Yoshida et al, 1998). In the same study, homozygous deletion of the *ANA* gene was found in a human non-small cell lung carcinoma (NSCLC), whereas no mutation of the gene was detected in primary NSCLCs. Given the high frequency of LOH in this region, we hypothesized that the *ANA* gene is one of the target genes for the development of human OSCC.

In the current study, we performed a polymerase chain reaction (PCR)-based LOH assay in the region spanning the *ANA* locus, PCR-single strand conformational polymorphism (PCR-SSCP) analysis, direct DNA sequencing and reverse transcription-PCR (RT-PCR) to examine the role of genetic alterations of the *ANA* gene in OSCCs. In addition, the level of *ANA* gene mRNA expression in OSCC-derived cell lines treated with or without a DNA methylating chemical (5-aza-2′-deoxycytidine) was assessed to determine if the transcriptional silencing of the gene is linked to promoter hypermethylation.

### MATERIALS AND METHODS

### Tumour specimen preparation and DNA/RNA extraction

63 pairs of tumour and corresponding normal oral mucosa specimens were obtained at the time of surgical resection between 1995 and 1999 at Chiba University Hospital and Tokyo Dental College Hospital. Informed consent was obtained from all patients and also from the families of the patients. The resected tissues were divided into 2 segments; one was frozen immediately after carefully removal from the surrounding normal tissues and stored at –80˚C until extraction of DNA or RNA, and the other was fixed in

*Received 2 August 2000*
*Revised 28 November 2000*
*Accepted 30 November 2000*

*Correspondence to:* H Tanzawa

10% formalin for pathologic diagnosis. Histopathologic diagnosis was performed according to the International Classification of Tumours (Wahi, 1971) by the Department of Pathology, Chiba University School of Medicine. Clinicopathologic staging was determined by the TNM staging system (Hermanek and Sobin, 1987). All patients had histologically confirmed SCC of the oral cavity and the tumour samples for DNA extraction were checked to ensure that they consisted of more than 80% tumour within the specimens. Genomic DNAs were extracted from powdered frozen tumour and corresponding normal tissues by proteinase K digestion and phenol-chloroform extraction, and then precipitated with ethanol (Maniatis et al, 1982).

Total RNA was extracted from 20 pairs of primary OSCC specimens and their adjacent normal epithelial tissues using an

SV Total RNA Isolation System (Promega, Madison, WI, USA) according to the manufacturer's protocol. Concentrations of DNA or RNA were estimated by a spectrophotometric method. The samples were stored at –80˚C until use.

### DNA analysis of microsatellite repeat polymorphism

7 microsatellite markers flanking the ANA locus were used to test for LOH. The primer sequences of the microsatellite repeat polymorphisms were obtained from the GenBank Sequence Database. Primers were isotopically end-labelled with [$\gamma$-$^{32}$P]-adenosine triphosphate (Amersham Pharmacia Biotech, Uppsala, Sweden), and PCR amplification was carried out in a final volume of 10 µl as described previously (Uzawa et al, 1996). PCR products from tumour and corresponding control DNAs were loaded in parallel on 5% polyacrylamide gel containing 7 M urea and visualized by autoradiography. Duplicate examinations were performed to confirm LOH on 21q. LOH for tumour DNA samples was assessed by scanning densitometry and analysed by National Institutes of Health (NIH) software (Image version 1.62, Dr. W. Rasband, NIH, Bethesda, MD, USA). The intensities of the signals in tumour DNA were compared with those of the corresponding normal DNA. We used Fisher's exact test to analyse for significance of differences in frequencies of LOH between TNM staging and tumour differentiation. The accepted level was $P < 0.05$.

### PCR-SSCP and direct DNA sequence analyses

To screen for sequence variations of the *ANA* gene among patients with OSCC, PCR-SSCP analysis was performed as described previously (Uzawa et al, 1995). Exons from 2 to 5 of the *ANA* gene were amplified with the specific primers according to Kohno et al (1998). Mobility shifted bands identified by SSCP analysis were excised, eluted from the gels, and re-amplified by PCR using the initial PCR primers. The PCR fragments were purified and sequenced using a cycle-sequencing method as described previously (Uzawa et al, 1995).

### Evaluation of mRNA expression of the *ANA* gene

To create first-strand cDNA for the *ANA* gene, 1.5 µg of total RNA was used for RT. The reaction was performed using a Ready-To-Go T-Primed First-Strand Kit (Amersham Pharmacia Biotech). A 10% portion of the cDNA was amplified by PCR. The specific primers for the *ANA* gene were synthesized based on the previously published sequence (Yoshida et al, 1998). To amplify the cDNA for the *ANA* gene (GenBank accession #D64110), the primer sequences were: Ana-F1 5′-GAATCACTATCCTCCTC-CTGT- 3′; Ana-R1 5′- GATGGTTTGGCCCATCTAAC-3′.

cDNA preparations were done in the presence and absence of RT, the latter acting as a control for contaminating genomic DNA from which fragments of the pseudogene can be amplified with these primers. PCR amplification of cDNA for the *ANA* gene was performed in 12.5 µl of PCR Master Mix (Roche Molecular Biochemicals, Mannheim, Germany), 1 µl of the cDNA obtained from the RT reaction, 1 µl of each of the specific primers (0.5 µg µl$^{-1}$) described previously and 10.5 µl of water. After amplification, an aliquot of the PCR product (271 bp length) was separated on a 3% TAE-agarose gel, stained with ethidium bromide. The density of the ethidium bromide-stained bands was quantitated using NIH image software. The results were normalized as a ratio of each specific mRNA signal to the glyceraldehyde-3-phosphate dehydrogenase gene (*GAPDH*) signal within the same RNA sample. The expression ratio of the tumour was divided by that of corresponding normal tissue to obtain the gene conservation rate. When the conservation rate of a given specimen was less than 1.0, this denoted reduced gene expression. Reproducibility was confirmed by processing all samples at least twice.

### Cell lines and cell culture

The following OSCC-derived cell lines were analysed: SAS, HSC-2, HSC-3, HSC-4, Ca9-22, Ho-1-u-1, Ho-1-N-1, SCC4 (obtained from Human Science Research Resources Bank, Osaka, Japan), and OK-92 (established from carcinoma of the tongue in our department). All OSCC-derived cell lines were grown in RPMI-1640 medium with 10% fetal bovine serum and 50 units ml$^{-1}$ penicillin and streptomycin. When cells reached confluence, they were washed twice in cold phosphate buffered saline (PBS) and total RNA was isolated with the same kit used for OSCC tumours. RT-PCR was performed to examine the state of *ANA* gene expression as mentioned previously.

### 5-aza-2′-deoxycytidine treatment

To assess re-activation of the gene expression, the cells were treated with different concentrations (0 and 2 µM) of the DNA methyltransferase inhibitor, 5-aza-2′-deoxycytidine, as described previously (Timmermann et al, 1998). On day 5, cells were washed with PBS and grown for an additional 10 days without the demethylating chemical. After that, the cells were harvested, and total RNA was extracted and the expression of the *ANA* gene was evaluated by the RT-PCR as described previously.

### RESULTS

### Deletion mapping around the ANA locus

A deletion map flanking the ANA locus was created for 126 DNA samples obtained from oral tumours and normal tissues (Figure 1). Of 63 cases that were informative for at least 1 of the loci, 32 (50.8%) showed allelic deletions. The frequencies of LOH for the 7 markers are listed in Table 1. The most frequent allelic losses were identified at markers D21S369 (37%) at 21q and D21S236 at 21q (38%). These microsatellite markers were mapped to the centromeric region of the ANA locus (Table 1). The results of LOH analyses in the 32 LOH-positive cases are summarized in the

*© 2001 Cancer Research Campaign*

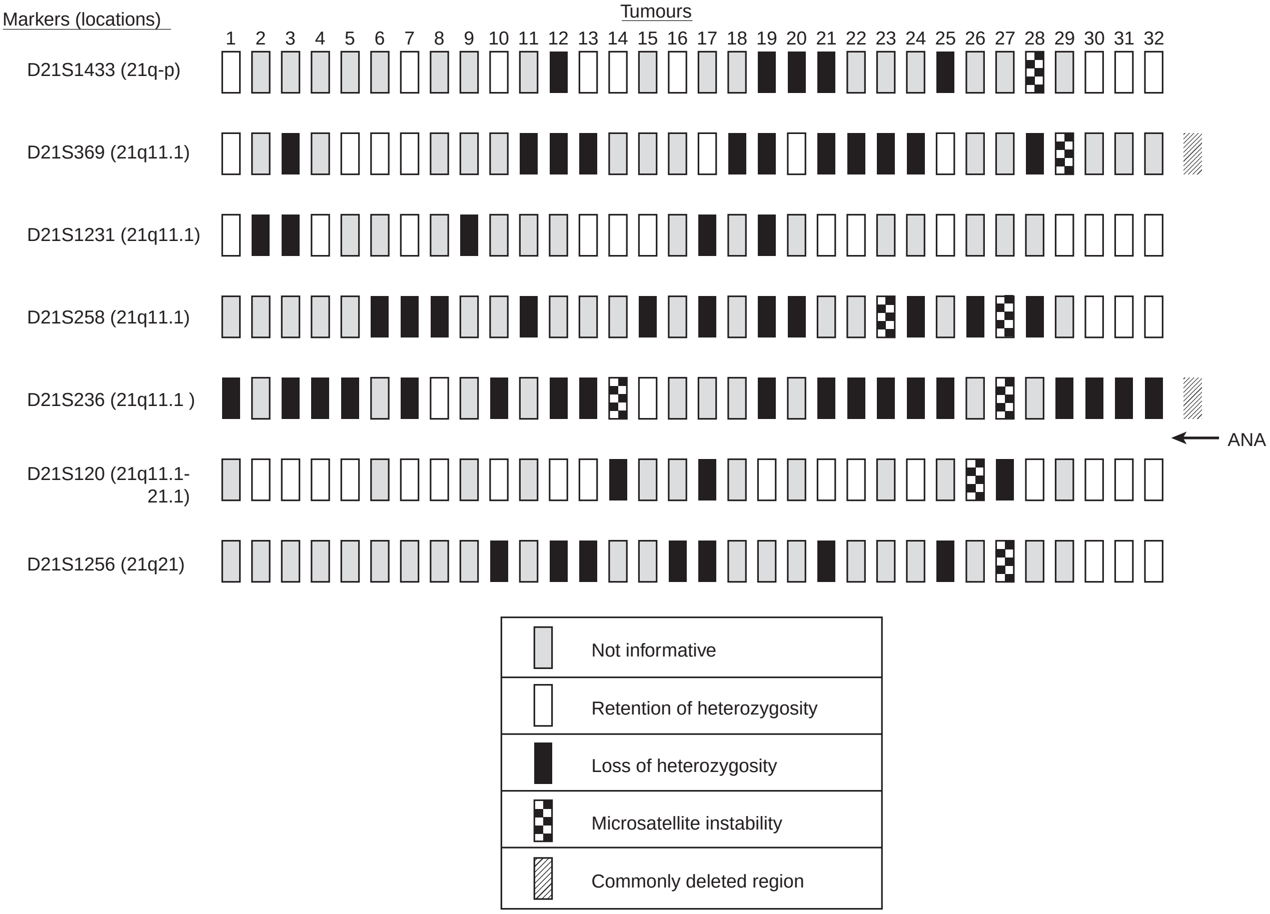

**Figure 1** A detailed deletion map of the area around the *ANA* locus in human OSCC. 32 cases showed LOH at one or more loci. The case number is shown above each column, and 7 microsatellite markers are indicated on the left. 2 distinct commonly deleted regions are shown at markers D21S369 and D21S236 in the centromeric location of the *ANA* locus deletion map in Figure 1 and representative results are shown in Figure 2.

In addition, we compared our results with the clinicopathologic factors. A number of sites displaying LOH at 21q could be detected in early-stage lesions; the frequencies of LOH tended to be higher in later clinical stages, but no statistical correlation was observed. Furthermore, we failed to detect any relation between the number of deletions per specimen and the grade of differentiation.

**Table 1** Loss of heterozygosity at 7 microsatellite regions on 21q in primary OSCCs

| Marker | Cytogenetic location | Frequency of allelic loss (%) (LOH / informative cases) |
|---|---|---|
| Centromere ↑ | | |
| D21S1433 | 21p–q | 15 (6/40) |
| D21S369 | 21q11.1 | 37 (11/30) |
| D21S1231 | 21q11.1 | 17 (5/30) |
| D21S258 | 21q11.1 | 22 (11/50) |
| D21S236 | 21q11.1 | 38 (18/48) |
| D21S120 | 21q11.1–21 | 11 (3/28) |
| *ANA* | 21q11.2–21.1 | – |
| D21S1256 | 21q21 | 14 (7/50) |
| Telomere ↓ | | |

### Mutation status of the *ANA* gene

By PCR-SSCP analysis, bands with altered mobility were detected in 6 cases. Among them, 5 cases showed normal sequence polymorphisms with a single base change (GGA/GAA at codon 40) (data not shown). In contrast, a single nucleotide substitution from GAG to GCG at codon 25, resulting in an amino acid replacement of glutamine with alanine, was found in tissue sample 21 (Figure 3). The tumour of this patient was aggressive and classified as clinical stage IV.

### Frequency of decreased levels of *ANA* mRNA in primary OSCCs and in OSCC-derived cell lines

The expression levels of *ANA* mRNA were examined in 20 paired samples of primary OSCC tissues and matched adjacent normal oral tissues, and in 9 OSCC-derived cell lines by RT-PCR analysis. All normal tissues revealed a 271-bp *ANA* mRNA transcript, which was the respective length of the PCR product. Among 20 cases tested, 12 oral tumours showed absent or significantly reduced expression of the *ANA* gene (Table 2). The *ANA* gene expression rate ranged from 0 to 0.25. Representative results are summarized in Figure 4A. 11 of 12 tumours with suppressed *ANA* gene expression were stage IV tumours. 8 of 9 cell lines had

 *© 2001 Cancer Research Campaign*

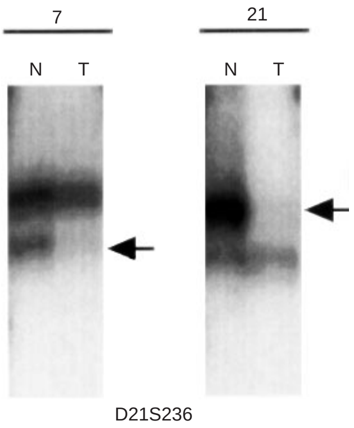

**Figure 2** Selection of tumours showing LOH (arrows) at 21q11.1 (D21S236 locus) (cases 7 and 21). Case numbers are shown above. N, corresponding normal tissue; T, tumour tissue

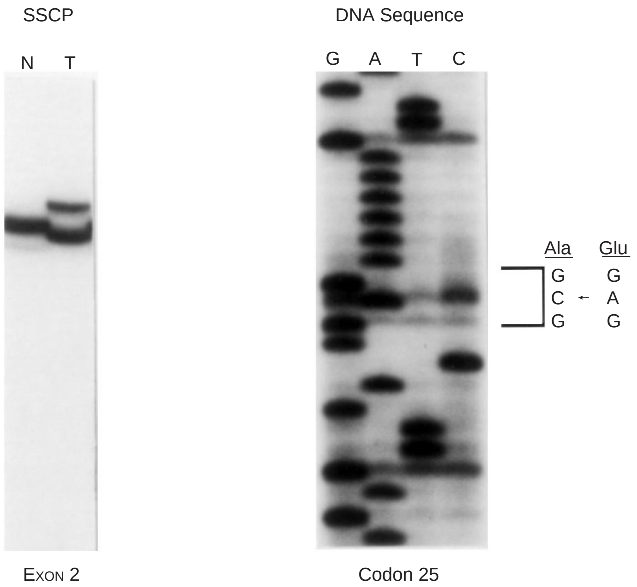

**Figure 3** Evidence for 2 mutational events in the *ANA* gene of a patient with OSCC (case 21). Left panel, PCR-SSCP analysis for exon 2 of the *ANA* gene. Abnormality mobility shifts are evident in lane T (tumour) when compared with lane N (normal tissue); right panel, identification of a nucleotide substitution in the tumour DNA sample. The 25th codon in the sample changed from GAG (glutamine) to GCG (alanine)

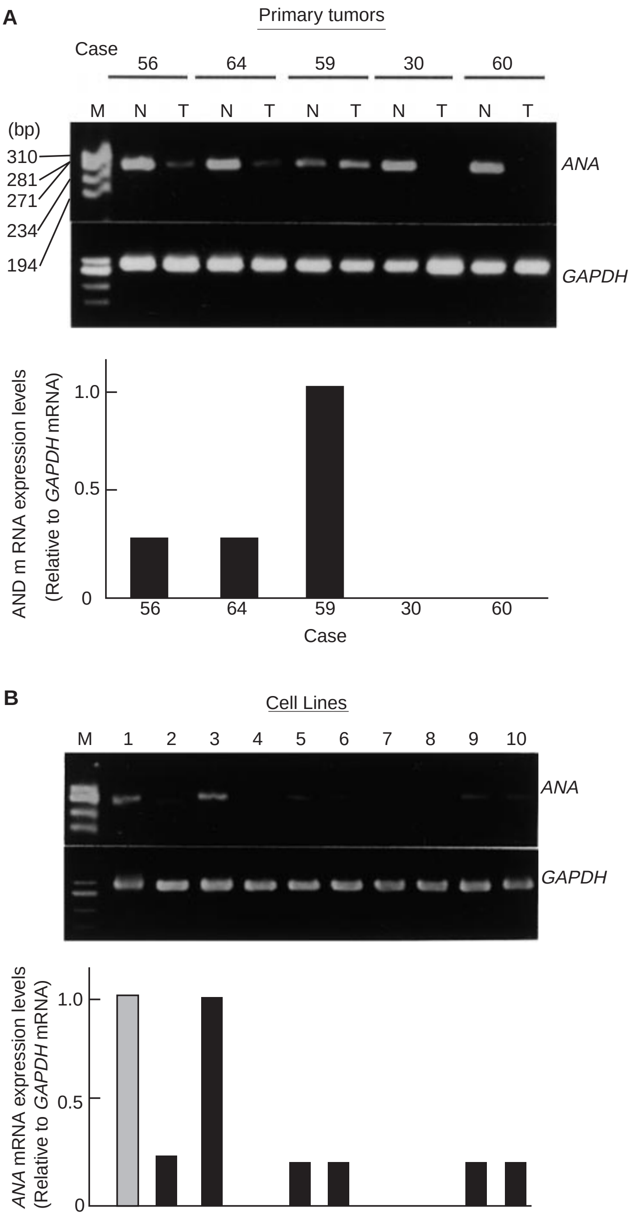

**Figure 4** RT-PCR analysis of freshly resected tissue samples (tumours, T; corresponding normal tissues, N) for the *ANA* gene (**A**) and OSCC-derived cell lines (**B**) (1, normal tissue; 2, SAS; 3, HSC-2; 4, HSC-3; 5, HSC-4; 6, Ca9-22; 7, OK-92; 8, Ho-1-u-1; 9, Ho-1-N-1; 10, SCC-4). Each lower panel indicates quantitation of the *ANA* gene RT-PCR products normalized to the level of *GAPDH* mRNA

decreased *ANA* mRNA expression compared to the average expression level seen in 20 independently analysed normal oral tissues (Figure 4B).

### Re-activation of *ANA* gene expression by the DNA methyltransferase inhibitor (5-aza-2′-deoxycytidine)

Of the cell lines with reduced or absent *ANA* gene mRNA expression, 4 (SAS, HSC-4, Ca9-22, and OK-92) showed significantly increased expression or re-expression of the gene after treatment 5-aza-2′-deoxycytidine. It is noteworthy that these cells re-activating ANA had a senescence-like state with significant up-regulation of the *ANA* gene. A representative result of OSCC-derived cell line, HSC-4, is shown in Figure 5.

### DISCUSSION

Allelic deletions on human 21q have been reported in several types of human cancers, such as ovarian cancer (Cliby et al, 1993), lung cancer (Sato et al, 1994; Kohno et al, 1998), oesophageal cancer (Aoki et al, 1994), and renal cell carcinoma (Schwerdtle et al, 1996). Recently, Sakata et al (1997) constructed a detailed deletion map of 21q in stomach cancer and found 2 commonly deleted regions at markers D21S1254 and D21S1456. More recently, Ohgaki et al (1998) identified a commonly deleted region spanning 6 centi-morgans at 21q21 in breast cancer, which overlapped the proximal commonly deleted region in gastric cancer. These

*© 2001 Cancer Research Campaign* 

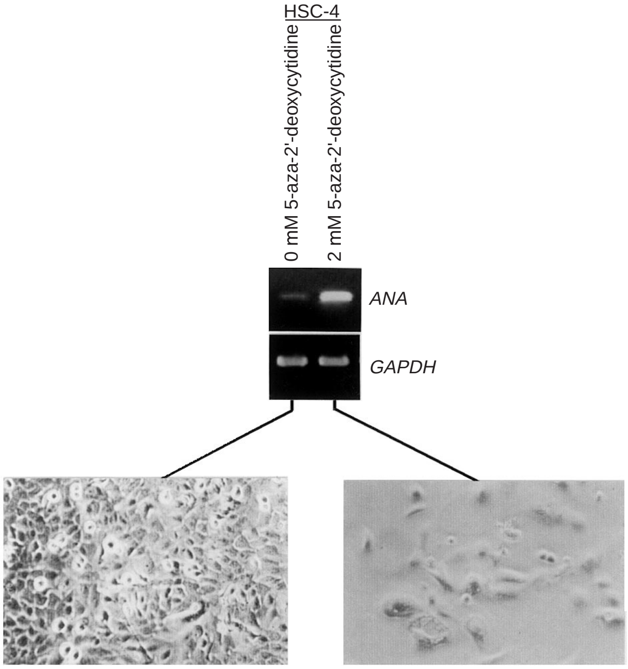

**Figure 5** A representative results of re-activation of the *ANA* gene in OSCC-derived cell line (HSC-4) after treatment with 5-aza-2′-deoxycytidine. The OSCC cell line was treated for 5 days with or without 2 μM 5-aza-2′-deoxycytidine and cultured for an additional 10 days without the demethylating agent. Note that significant up-regulation of the *ANA* gene mRNA and that induction of a senescence-like state is seen in 5-aza-2′-deoxycytidine treated HSC-4 cell line. *GAPDH* gene mRNA signal was used for the internal control of the analysis. Original magnification ×400

observations suggest that more than one TSG specific to several types of human malignancies may exist on 21q. In our previous study, 3 potential TSG loci were identified on 21q in OSCCs (Yamamoto et al, 1999). Of them, the most frequently deleted region was identified on 21q11.1, which is near the *ANA* gene locus. In the present study, we extended these initial observations and identified 2 novel commonly deleted regions that are distinct from and centromeric to the ANA locus (Table 1, Figure 1). Interestingly, these regions are clearly different from those of commonly deleted regions identified in gastric or breast cancer. Therefore, the data suggest that there may be new target genes at 21q11.1 and that the genes may be specifically associated with the pathogenesis of human OSCCs. On the other hand, our failure to find any correlation between 21q LOH and clinicopathologic characteristics, such as tumour size, lymph node status, clinical stage, and histologic grade, might suggest that inactivation of the unknown TSGs on 21q is an early event in carcinogenesis in a subgroup of OSCCs but with little or no influence on further tumour progression.

The *ANA* gene, mapped on human chromosome 21q11.2–q21.1, is a member of an anti-proliferative gene family, based on the fact that it can inhibit the proliferative activity in NIH3T3 cells (Yoshida et al, 1998). A homozygous deletion of the gene was found in a human non-small cell lung cancer cell line, suggesting that the gene is a candidate TSG (Kohno et al, 1998). In our previous study, frequent allelic loss was identified on chromosome arm 21q21 that includes the *ANA* gene locus in primary OSCCs (Yamamoto et al, 1999). Thus, we hypothesized that loss of function of the *ANA* gene could contribute to the tumorigenesis of OSCC. Almost half of the oral tumours analysed in the present study showed suppressed expression of the *ANA* gene (Table 2, Figure 4A), suggesting that transcriptional silencing through the gene expression might occur. However, an intragenic mutation of the *ANA* gene was observed in only one advanced case, while others showed no mutation affecting gene function (Figure 2). It has been widely believed that 2 mutational hits (mutation and LOH) are required for the inactivation of a TSG. However, there is considerable recent evidence that abnormal methylation at the promoters of TSGs is a novel mechanism for the suppression of these genes activity, which is defined as a third pathway (Jones and

**Table 2** Summary of the molecular status of the *ANA* gene and clinicopathologic features in primary OSCCs

| Tumour No. | Tumour differentiation | Stage | Site | *ANA* mRNA expression level | 21q-LOH |
|---|---|---|---|---|---|
| 52 | W | II | Tongue | Normal | LOH |
| 55 | P | II | Tongue | Normal | ROH |
| 14 | M | II | Buccal mucosa | Normal | LOH |
| 63 | W | IV | Gingiva | Normal | NI |
| 56 | W | IV | Tongue | Reduced | ROH |
| 57 | M | I | Lip | Normal | ROH |
| 64 | W | IV | Gingiva | Reduced | NI |
| 58 | M | IV | Gingiva | Normal | ROH |
| 59 | W | I | Tongue | Normal | ROH |
| 60 | W | III | Tongue | Negative | ROH |
| 61 | W | IV | Buccal mucosa | Negative | ROH |
| 25 | W | IV | Gingiva | Negative | LOH |
| 30 | P | IV | Tongue | Negative | LOH |
| 31 | M | III | Tongue | Reduced | LOH |
| 32 | W | II | Oral floor | Normal | LOH |
| 62 | M | III | Tongue | Negative | ROH |
| 54 | W | IV | Gingiva | Negative | ROH |
| 43 | P | IV | Tongue | Negative | ROH |
| 49 | M | III | Tongue | Negative | LOH |
| 45 | W | IV | Gingiva | Reduced | ROH |

W, well-differentiated OSCC; M, moderately differentiated OSCC; P, poorly differentiated OSCC; LOH, loss of heterozygosity; ROH, retention of heterozygosity; NI, not informative.

© 2001 Cancer Research Campaign

Laird, 1999). Therefore, we hypothesized that down-regulation of the gene expression could be linked to this pathway. Because the promoter region of the *ANA* gene has not been identified, we tested whether treatment with a demethylating agent (5-aza-2′-deoxycytidine) would lead to increased steady levels of the *ANA* gene in OSCC-derived cell lines. The *ANA* gene was re-activated in 4 of 8 OSCC-derived cell lines after treatment with the DNA demethylation agent, which showed down-regulation of the gene (Figure 5), indicating that the promoter region of these 4 cell lines is highly methylated. Therefore, our results suggest that the *ANA* gene might be silenced by an epigenetic mechanism involving aberrant DNA methylation. It would be interesting to clarify the methylation status in human OSCC in future investigations. In addition, it is worthwhile to mention that down-regulation of the *ANA* gene was observed more frequently in stage IV tumours than in earlier stage tumours (Table 2), suggesting that the expression of the *ANA* gene is associated with the progression of OSCC.

In conclusion, our observations suggest that repression of *ANA* gene expression frequently accompanies tumour development in OSCC, while *ANA* mutation is very rare. In addition, the deletion map generated by the present study has provided additional evidence for the presence of new TSGs by showing novel LOH sites at 21q11.1. However, whether the 21q-LOH and/or inactivation of the *ANA* gene could be an important predictor of prognosis and disease outcome needs to be clarified by further molecular epidemiologic studies.

### ACKNOWLEDGEMENTS

The authors would like to thank Ms Lynda C Charters for proof-reading this manuscript. This work was supported by Research Grants from the Ministry of Education, Science and Culture, Japan.

### REFERENCES

Ah-See KW, Cooke TG, Pickjord IR, Soutar D and Balmain A (1994) An allelotype of squamous cell carcinoma of the head and neck using microsatellite markers. *Cancer Res* **54**: 1617–1621

Aoki T, Mori T, XiQun D, Nishira T, Matsubara T and Nakamura Y (1994) Allelotype study of esophageal carcinoma. *Genes Chromosomes Cancer* **10**: 177–182

Chiba I, Shindoh M, Yasuda M, Yamazaki Y, Amemiya A, Sato Y, Fujinaga K, Notani K and Fukuda H (1996) Mutations in the p53 gene and human papillomavirus infection as significant prognostic factors in squamous cell carcinomas of the oral cavity. *Oncogene* **18**: 1663–1668

Cliby W, Ritland S, Hartmann L, Dodson M, Halling KC, Keeney G, Podratz KC and Jenkins RB (1993) Human epithelial ovarian cancer allelotype. *Cancer Res* **53**: 2393–2398

Fearon ER (1998) Tumor suppressor genes. In: *The Genetic Basis of Human Cancer*, Vogelstein B and Kinzler KW (eds), pp. 229–236. McGraw-Hill: New York

Field JK, Kiaris H, Risk JM, Tsiriyotis C, Adamson R, Zoumpourlis V, Rowley H, Taylor K, Whittaker J, Howard P, Beirnie JC, Gosney JR, Woolgar J, Vaughan ED, Spandidos DA and Jones AS (1995) Allelotype of squamous cell carcinoma of the head and neck: fractional allele loss correlates with survival. *Br J Cancer* **72**: 1180–1188

Heinzel PA, Balaram P and Bernard HU (1996) Mutations and polymorphisms in the p53, p21 and p16 genes in oral carcinomas of Indian betel quid chewers. *Int J Cancer* **68**: 420–423

Hermanek P and Sobin LH (1987) *UICC, TNM Classification of Malignant Tumours*, 4th ed. pp. 16–18. Springer Verlag: Berlin

Ishwad CS, Ferrell RE, Rossie KM, Appel BN, Johnson JT, Myers EN, Law JC, Srivastava S and Gollin SM (1995) Microsatellite instability in oral cancer. *Int J Cancer (Pred Oncol)* **64**: 332–335

Jones PA and Laird PW (1999) Cancer epigenetics comes of age. *Nature Genet* **21**: 163–167

Kohno T, Kawanishi M, Matsuda S, Ichikawa H, Takada M, Ohki M, Yamamoto T and Yokota J (1998) Homozygous deletion and frequent allelic loss of the 21q11.1–q21.1 region including the ANA gene in human lung carcinoma. *Genes Chromosomes Cancer* **21**: 236–243

Largery JS, Meltzer SJ, Sauk JJ, Hebert CA and Archibald (1994) Loss of heterozygosity involving the APC gene in oral squamous cell carcinoma. *Oral Surg Oral Med Oral Path* **77**: 260–263

Maestro R, Gasparotto D, Vukosavljevic T, Barzan L, Sulfaro S and Boiocchi M (1993) Three discrete regions of deletion at 3p in head and neck cancers. *Cancer Res* **53**: 5775–5779

Maniatis T, Fritsch EF and Sambrook J (1982) *Molecular Cloning, a Laboratory Manual* ed. 1. pp. 280–281, Cold Spring Harbor Laboratory Press: Cold Spring Harbor, NY

Miracca EC, Kowalski LP and Nagai MA (1999) High prevalence of p16 genetic alterations in head and neck tumours. *Br J Cancer* **81**: 677–683

Ogawara K, Miyakawa A, Shiiba M, Uzawa K, Watanabe T, Wang X-L, Sato T, Kubosawa H, Kondo Y and Tanzawa H (1998) Allelic loss of chromosome 13q14.3 in human oral cancer: correlation with lymph node metastasis. *Int J Cancer* **79**: 312–317

Ohgaki K, Iida A, Kasumi F, Sakamoto G, Akimoto M, Nakamura Y and Emi M (1998) Mapping of a new target region of allelic loss to a 6-cM interval at 21q21 in primary breast cancers. *Genes Chromosomes Cancer* **23**: 244–247

Sakata K, Tamura G, Nishizuka S, Maesawa C, Suzuki Y, Iwaya T, Terashima M, Saito K and Satodate R (1997) Commonly deleted regions on the long arm of chromosome 21 in differentiated adenocarcinoma of the stomach. *Genes Chromosomes Cancer* **18**: 318–321

Sato S, Nakamura Y and Tsuchiya E (1994) Difference of allelotype between squamous cell carcinoma and adenocarcinoma of the lung. *Cancer Res* **54**: 5652–5655

Schwerdtle RF, Storkel S, Neuhaus C, Brauch H, Weidt E, Brenner W, Hohenfellner R, Huber C and Decker HJ (1996) Allelic losses at chromosomes 1p, 2p, 6p, 10p, 13q, 17p, and 21q significantly correlate with the chromophobe subtype of renal cell carcinoma. *Cancer Res* **56**: 2927–2930

Scully C, Field JK (1997) Genetic aberrations in squamous cell carcinoma of the head and neck (SCCHN), with reference to oral carcinoma (Review). *Int J Oncol* **10**: 5–21

Timmermann S, Hinds PW and Münger K (1998) Re-expression of endogenous $p16^{ink4a}$ in oral squamous cell carcinoma lines by 5-aza-2′-deoxycytidine treatment induces a senescence-like state. *Oncogene* **17**: 3445–3453

Uzawa K, Yoshida H, Suzuki H, Tanzawa H, Shimazaki J, Seino S and Sato K (1994) Abnormalities of the adenomatous polyposis coli gene in human oral squamous cell carcinoma. *Int J Cancer* **58**: 814–817

Uzawa K, Suzuki H, Yokoe H, Tanzawa H and Sato K (1995) Mutational state of *p16/CDKN2* and *VHL* genes in squamous-cell carcinoma of the oral cavity. *Int J Oncol* **7**: 895–899

Uzawa K, Suzuki H, Komiya A, Nakanishi H, Ogawara K, Tanzawa H and Sato K (1996) Evidence for two distinct tumor-suppressor gene loci on the long arm of chromosome 11 in human oral cancer. *Int J Cancer* **67**: 510–514

Wang X-L, Uzawa K, Imai FL and Tanzawa H (1999) Localization of a novel tumor suppressor gene associated with human oral cancer on chromosome 4q25. *Oncogene* **18**: 823–825

Wahi PN (1971) Histological typing of oral and oropharyngeal tumours. In: *International Histological Classification of Tumours, No. 4*. World Health Organization: Geneva

Wu CL, Roz L, Sloan P, Read AP, Holland S, Porter S, Scully C, Speight PM and Thakker N (1997) Deletion mapping defines three discrete areas of allelic imbalance on chromosome arm 8p in oral and oropharyngeal squamous cell carcinomas. *Genes Chromosomes Cancer* **20**: 347–353

Yamamoto N, Uzawa K, Miya T, Watanabe T, Yokoe H, Shibahara T, Noma H and Tanzawa H (1999) Frequent allelic loss/imbalance on the long arm of chromosome 21 in oral cancer: Evidence for three distinct tumor suppressor gene loci. *Oncol Rep* **6**: 1223–1227

Yoshida Y, Matsuda S, Ikematsu N, Kawamura-Tsuzuku J, Inazawa J, Umemori H and Yamamoto T (1998) *ANA*, a novel member of *Tob/BTG1* family, is expressed in the ventricular zone of the developing central nervous system. *Oncogene* **16**: 2687–2693

*© 2001 Cancer Research Campaign*
